# Upregulation of Orai Channels Contributes to Aging-Related Vascular Alterations in Rat Coronary Arteries

**DOI:** 10.3390/ijms241713402

**Published:** 2023-08-29

**Authors:** Javier Angulo, Argentina Fernández, Alejandro Sevilleja-Ortiz, Alberto Sánchez-Ferrer, Leocadio Rodríguez-Mañas, Mariam El Assar

**Affiliations:** 1 Servicio de Histología, Unidad de Investigación Cardiovascular (IRYCIS/UFV), Hospital Universitario Ramón y Cajal, 28034 Madrid, Spain; argentina.fernandez@salud.madrid.org (A.F.); alejandro.sevilleja@edu.uah.es (A.S.-O.); 2 Centro de Investigación Biomédica en Red de Fragilidad y Envejecimiento Saludable (CIBERFES), Instituto de Salud Carlos III, 28029 Madrid, Spain; leocadio.rodriguez@salud.madrid.org; 3 Fundación para la Investigación Biomédica, Hospital Universitario de Getafe, 28905 Getafe, Spain; asferrer@salud.madrid.org; 4 Servicio de Geriatría, Hospital Universitario de Getafe, 28905 Getafe, Spain

**Keywords:** vascular aging, coronary artery, Orai channel, endothelial function, arterial hypercontractility

## Abstract

Vascular territories display heterogeneous sensitivity to the impacts of aging. The relevance of the STIM/Orai system to vascular function depends on the vascular bed. We aimed to evaluate the contribution of the STIM/Orai system to aging-related vascular dysfunction in rat coronary circulation. Vascular function was evaluated according to myography in coronary arteries from young (three-month-old) and older (twenty-month-old) rats. The effects of aging and STIM/Orai inhibition on the contraction and relaxation of the coronary arteries and on the protein expression of STIM-1, Orai1, and Orai3 in these vessels were determined. Aging-related hypercontractility to serotonin and endothelin-1 in arteries from male rats was reversed by STIM/Orai inhibition with YM-58483 or by specifically blocking the Orai1 channel with Synta66. The inhibitory effects of Synta66 on coronary vasoconstriction were also observed in older female rats. YM-58483 relaxed serotonin- but not KCl-contracted arteries from males. STIM/Orai inhibition improved defective endothelial vasodilations in aged arteries, even in the presence of NO synthase and cyclooxygenase inhibitors, but not in KCl-contracted segments. YM-58483 significantly enhanced relaxations to calcium-activated potassium channel stimulation in aged vessels. Increased protein expression of Orai1 and Orai3 was detected in arterial homogenates and sections from older rats. Upregulation of the Orai channel contributes to aging-related coronary dysfunction, revealing a potential target in reducing CVD risk.

## 1. Introduction

Cardiovascular diseases (CVDs) are the leading cause of death among older subjects [1]. In fact, age is a main risk factor for CVD even in the absence of other traditional risk factors [2]. Vascular dysfunction not only underlies the development of CVD but may also condition the functional status of older people [3,4]. Although vascular function has been shown to be progressively compromised by aging in different vascular beds from animals and humans [5,6,7,8], differences exist at the chronological, qualitative, and quantitative levels with respect to the aging-related impact on vascular function depending on the vascular bed [7,8]. In this sense, the coronary vascular bed seems to be a vascular territory that is especially sensitive to the impact of aging in rats, displaying, when compared to other vessels, a deeper and earlier impairment of endothelial vasodilation, as well as a clearer enhancement of vasoconstriction [7]. Coronary dysfunction is a key contributor to CVDs such as heart failure [9]. However, the mechanisms leading to coronary dysfunction with aging are not yet fully elucidated.

Calcium homeostasis plays a key role in vascular function since it is fundamental to vasomotor responses, in addition to vascular remodeling [10]. Thus, alterations affecting calcium-regulating systems would have an impact on vascular function. Store-operated calcium entry (SOCE) allows for the recharging of intracellular calcium concentrations when reservoirs are empty. When calcium concentrations in the endoplasmic reticulum (ER) decrease, the sensor protein, stromal interaction molecule (STIM), in the ER membrane activates Orai channels located in the plasmatic membrane to allow for calcium entry. Two isoforms of STIM (STIM-1 and STIM-2) and three isoforms of Orai (Orai1, Orai2, and Orai3) have been described [11]. Some evidence suggest that STIM-2 is only relevant in the absence of STIM-1 [12] or that it is less effective than STIM-1 in activating calcium entry through an interaction with Orai [13]. On the other hand, there is scant evidence for the relevance of Orai2 in vascular physiology, while Orai1 and Orai3 have been shown to be activated in a STIM-independent way with relevance to vascular pathological situations [14]. In this sense, Orai1 and Orai3 were found to be upregulated in rat and human corpus cavernosum from men with diabetic erectile dysfunction [15]. In fact, we have previously found altered STIM/Orai calcium systems in the aged vasculature of rats and humans, including penile vascular tissue, as well as large and small vessels, but with significant differences detected among the different vascular beds [16,17]. However, to our knowledge, there is no information on the influence of aging on the STIM/Orai system in coronary circulation.

The aim of the present work was to evaluate the relevance of the STIM/Orai system in aging-related vascular alterations to rat coronary arteries. The effects of STIM/Orai modulation on contractile and relaxant responses in isolated coronary arterial segments, as well as the influence of aging on STIM-1, Orai1 and Orai3 protein expression in coronary vessels, were determined.

## 2. Results

### 2.1. The STIM/Orai System Contributes to Aging-Induced Hypercontractility in Rat Coronary Arteries

**Figure 1:**
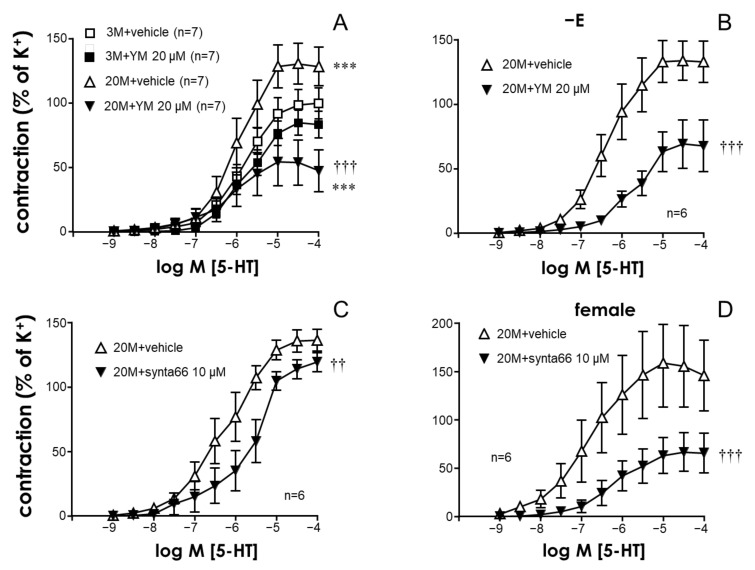
STIM/Orai inhibition reduces the aging-related enhancement of serotonin-induced contractions in rat coronary arteries. Panel (**A**) shows the effects of the treatment with the vehicle (0.2% DMSO) or the STIM/Orai inhibitor, YM-58483 (YM, 20 µM), on serotonin (5-HT, 1 nM to 100 µM)-induced contractions in coronary arteries from young (3-month-old, 3 M) and old (20-month-old, 20 M) rats. Panel (**B**) shows the effects of the vehicle (0.2% DMSO) or YM (20 µM) on 5-HT-induced contractions in coronary arteries from older rats where the endothelium was previously removed. The lower panels show the effects of the treatment with the vehicle (0.1% DMSO) or the specific Orai1 inhibitor, synta66 (10 µM), on 5-HT-induced contractions in coronary arteries from older male (**C**) and female (**D**) rats. Data are expressed as the mean± S.E.M. of the percentage of 125 mM K^+^-induced contraction. n indicates the number of animals used for the experiments. *** indicates *p* < 0.001 vs. 3 M, †† *p* < 0.01, ††† *p* < 0.001 vs. vehicle via two-factor ANOVA.

Older male rats displayed significantly higher weights than young male and older female rats (696.4 ± 12.5 g vs. 474.1 ± 10.4 g, *p* < 0.001, and vs. 379.7 ± 13.9 g, *p* < 0.001, respectively). However, no significant differences in the average internal diameters of coronary segments (361.6 ± 8.1 µm, 387.2 ± 8.1 µm, and 371.8 ± 31.7 µm, for young male, older male, and older female rats, respectively, *p* > 0.05) and in the contractions induced by 125 mM K^+^ (6.07 ± 0.27 mN, 6.54 ± 0.24 mN, and 5.01 ± 0.44 mN, for young male, older male, and older female rats, respectively, *p* > 0.05) were observed among arteries from different groups of animals. In contrast, aging was related to a significant increase in contractions induced by serotonin (5-HT) in the coronary arteries from male rats (Figure 1A). The treatment of vascular segments with the STIM/Orai inhibitor YM-58483 (20 µM) did not significantly modify contractile responses to 5-HT or ET-1 in arteries from young (3-month-old) animals but significantly depressed 5-HT contractions in coronary arteries from older (20-month-old) rats (Figure 1A). In fact, in the presence of the STIM/Orai inhibitor, contractile responses in coronary arteries from old rats were significantly reduced with respect to those obtained from vessels from young animals (Figure 1A). The inhibitory effects driven by STIM/Orai inhibition on older coronary artery contractions induced by serotonin seemed to be ascribed to the smooth muscle layer, since, after endothelium removal, these inhibitory effects remained significant (Figure 1B). Furthermore, a specific blocker of Orai1 channels, Synta66 (10 µM), was able to significantly reduce serotonin-induced contractions in coronary arteries from older rats (Figure 1C).

**Figure 2:**
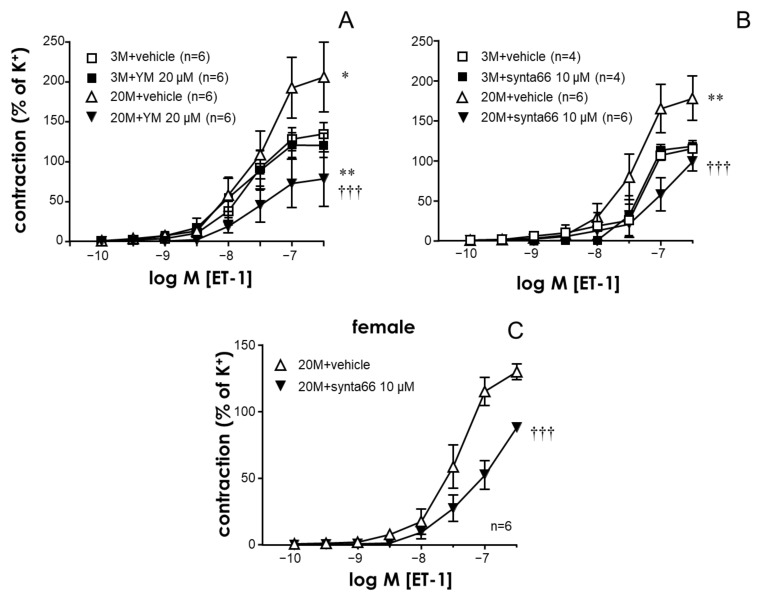
Aging-related enhancement of endothelin-1-induced contractions is reduced by STIM/Orai inhibition in rat coronary arteries. Panel (**A**) shows the effects of the treatment with the vehicle (0.2% DMSO) or the STIM/Orai inhibitor YM-58483 (YM, 20 µM) on endothelin-1 (ET-1, 0.1 nM to 0.3 µM)-induced contractions in coronary arteries from young (3-month-old, 3 M) and older (20-month-old, 20 M) rats. Panels (**B**,**C**) show the effects of the treatment with the vehicle (0.1% DMSO) and the specific Orai1 inhibitor, synta66 (10 µM), on ET-1-induced contractions in coronary arteries from young and older male rats (**B**) and from older female rats (**C**). Data are expressed as the mean ± S.E.M. of the percentage of 125 mM K^+^-induced contractions. n indicates the number of animals used for the experiments. * indicates *p* < 0.05, ** *p* < 0.01 vs. 3 M, ††† *p* < 0.001 vs. the vehicle according to two-factor ANOVA.

Contractions induced by the vasoactive peptide, endothelin-1 (ET-1), were also significantly enhanced in the coronary vascular segments from older rats (Figure 2A,B). Analogously to the phenomenon observed with serotonin, STIM/Orai inhibition with YM-58483 (20 µM) (Figure 2A) or specific Orai1 blockade with Synta66 (10 µM) (Figure 2B) did not significantly influence ET-1-induced contractions in arteries from young animals but markedly inhibited ET-1-induced contractions in coronary segments from older rats.

The impact of Orai inhibition on the contraction of older coronary arteries did not seem to be restricted to male rats, since the treatment with the Orai1 inhibitor Synta66 (10 µM) resulted in a significant reduction in 5-HT- and ET-1-induced contractions in coronary arteries from 20-month-old female rats (Figure 1D and Figure 2C).

### 2.2. STIM/Orai Inhibition Effects Are Lost under Smooth Muscle Depolarizing Conditions

Increasing the addition of KCl (10 to 120 mM) to the chambers caused concentration-dependent contractions of coronary arteries, which were not significantly different between young and older rats. These responses were unaffected by the presence of the STIM/Orai inhibitor YM-58483 (20 µM) in vessels from both younger and older animals (Supplementary Materials Figure S1).

**Figure 3:**
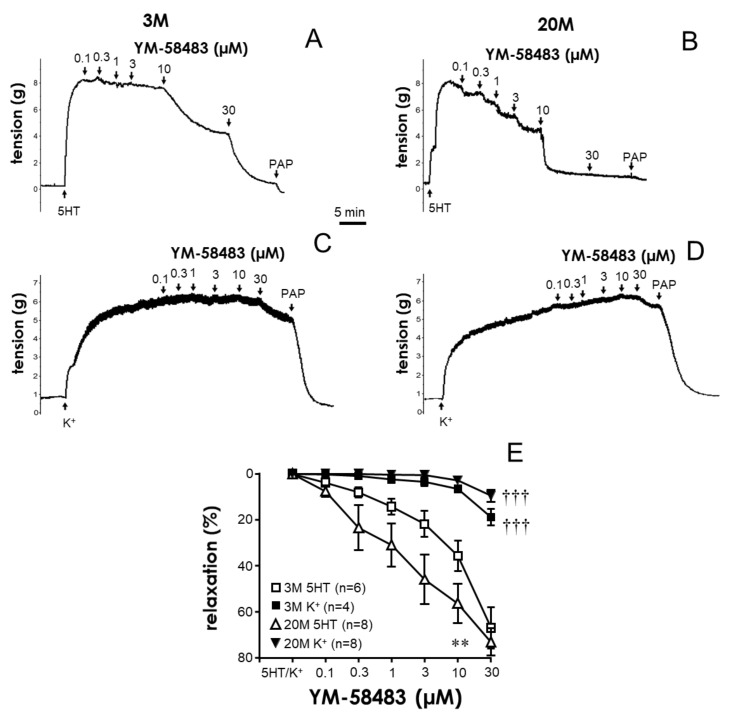
STIM/Orai inhibition caused the relaxation of serotonin-precontracted coronary arteries from male rats. Representative tracings of relaxations induced by the cumulative addition of YM-58483 (0.1 to 30 µM) in coronary arteries from young (3-month-old, 3 M) (**A**,**C**) and older (20-month-old, 20 M) (**B**,**D**) rats precontracted with serotonin (5 HT) (**A**,**B**) or KCl (K^+^) (**C**,**D**). PAP indicates papaverine (0.1 mM). Panel (**E**) shows the quantification of YM-58483-induced relaxations. Data are expressed as the mean ± S.E.M. of the percentage of maximum relaxation induced by papaverine at the end of the experiment. n indicates the number of animals used for the experiments. ** indicates *p* < 0.01 vs. 3 M, ††† *p* < 0.001 vs. 5 HT-contracted segments, assessed by two-factor ANOVA.

In coronary segments precontracted with serotonin, increasing the cumulative additions of YM-58483 (0.1 to 30 µM) resulted in concentration-dependent relaxations of the vessels (Figure 3A,B). These relaxation responses were significantly enhanced in coronary arteries from older rats (Figure 3E). However, when vascular segments were contracted with depolarizing concentrations of KCl (50 mM), relaxations to YM-58483 were almost completely blunted in coronary arteries from both young and older animals (Figure 3C–E).

### 2.3. STIM/Orai Inhibition Improved Endothelium-Dependent Vasodilation in Coronary Arteries from Older Rats

**Figure 4:**
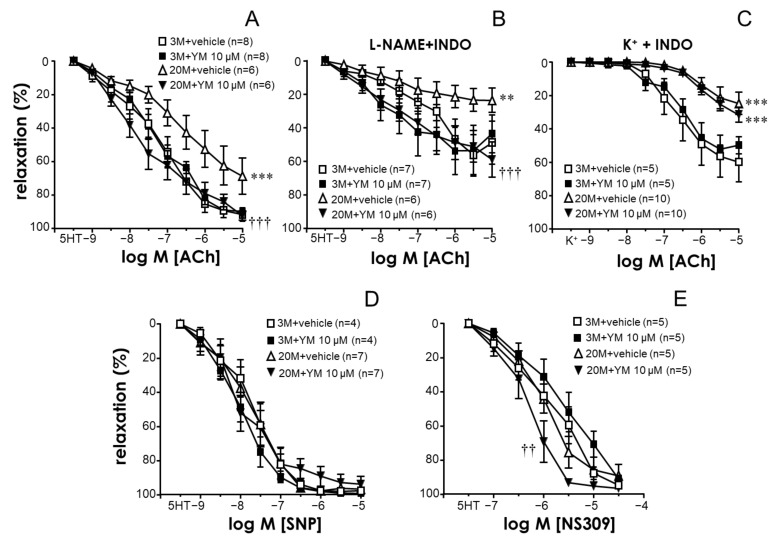
Aging-related impairment of endothelium-dependent vasodilation is alleviated by STIM/Orai inhibition in coronary arteries from male rats. The upper panels show the effects of the treatment with the vehicle (0.1% DMSO) or the STIM/Orai inhibitor YM-58483 (YM, 10 µM) on acetylcholine (ACh, 1 nM to 10 µM)-induced relaxation in coronary arteries from young (3-month-old, 3 M) and older rats (20-month-old, 20 M) contracted with serotonin (5 HT, 1–10 µM) under control conditions (**A**) or after treatment with the NO synthase inhibitor L-NAME (100 µM) and the cyclooxygenase inhibitor indomethacin (10 µM) (**B**), as well as in those contracted with KCl (K^+^, 50 mM) and treated with indomethacin (**C**). The lower panels show the effects of the treatment with the vehicle or YM on relaxations induced by the NO donor sodium nitroprusside (SNP, 1 nM to 10 µM) (**D**), and the SK_Ca_ and IK_Ca_ stimulator NS309 (0.1 to 30 µM) (**E**), in coronary arteries from 3 M and 20 M rats contracted with 5 HT. Data are expressed as the mean ± S.E.M. of the percentage of maximum relaxation induced by papaverine (0.1 mM) at the end of the experiment. n indicates the number of animals used for the experiments. ** indicates *p* < 0.01, *** *p* < 0.001 vs. 3 M, †† *p* < 0.01, ††† *p* < 0.001 vs. vehicle according to two-factor ANOVA.

When added to serotonin-contracted coronary segments, acetylcholine (ACh, 1 nM to 10 µM) caused endothelium-dependent relaxations. These relaxations were absent in endothelium-denuded segments (Supplementary Materials Figure S2A). The vasodilatory capacity of ACh was impaired in coronary arteries from older rats (Figure 4A). The treatment of older arteries with YM-58483 (10 µM) produced a significant improvement in ACh-induced relaxations, yielding responses not significantly different to those obtained in young vessels. Treatment with YM-58483 did not significantly modify endothelial relaxations in segments from young animals (Figure 4A).

Relaxation of the coronary segments by ACh in the presence of the NO synthase inhibitor L-NAME (100 µM) and the cyclooxygenase inhibitor indomethacin (10 µM) was also significantly impaired by aging. This impairment was reversed by treating older vascular segments with the STIM/Orai inhibitor, YM-58483 (10 µM). As was observed in control conditions, YM-58483 failed to significantly influence ACh-induced relaxations in coronary arteries from young animals after L-NAME and indomethacin treatments (Figure 4B). In contrast, YM-58483 was unable to significantly modify the ACh-induced relaxation of coronary arteries from older or young rats when the vessels were contracted with depolarizing 50 mM KCl and treated with indomethacin, despite the fact that, under these conditions, the vasodilatory responses were significantly impaired by aging (Figure 4C).

The potentiating effects of YM-58483 on endothelial vasodilation cannot be simply attributed to a potential effect on precontraction of 5-HT, since the effect of YM-58483 at 10 µM on the precontraction level in coronary arteries from old rats did not reach statistical significance either in terms of absolute values (4.68 ± 0.47 mN vs. 5.38 ± 1.03 mN for YM-58483 and the vehicle, respectively, *n* = 12, *p* = 0.8243) or in terms of the percentage of K^+^-induced contractions (71.89 ± 7.26% vs. 79.55 ± 6.67% for YM-58483 and the vehicle, respectively, *n* = 12, *p* = 0.3820).

Relaxations induced by the NO donor sodium nitroprusside (SNP, 1 nM to 10 µM) in rat coronary arteries were not significantly influenced by treatment with the STIM/Orai inhibitor, YM-58438 (10 µM), irrespective of whether they were obtained from young or older animals (Figure 4D). In contrast, relaxations driven by the stimulator of small- and intermediate-conductance calcium-activated potassium channels (SK_Ca_ and IK_Ca_), NS309 (0.1 to 30 µM), were potentiated by the treatment with YM-58483, but only in coronary segments from older animals (Figure 4E).

### 2.4. Functional Effects of STIM/Orai Inhibition Are Associated with the Increased Expression of Orai Channels in Coronary Arteries from Older Rats

**Figure 5:**
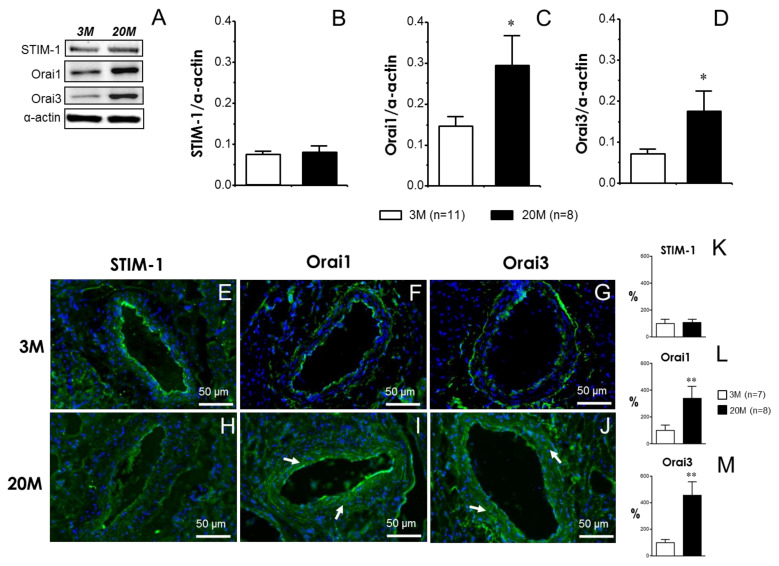
Orai1 and Orai3 channels are upregulated in rat coronary arteries from older male rats. Panel (**A**) shows representative immunoblots for the detection of STIM-1, Orai1, and Orai3, and corresponding α-actin in coronary artery homogenates from young (3-month-old, 3 M) and older rats (20-month-old, 20 M). The right panels show the quantification of the expression assays for STIM-1 (**B**), Orai1 (**C**), and Orai3 (**D**). Data are expressed as the mean ± S.E.M. of STIM-1, Orai1, and Orai3 band intensities normalized by respective α-actin band intensities. n indicates the number of animals from which the tissues were collected for the experiments. * indicates *p* < 0.05 vs. 3 M according to the Mann–Whitney U test. The lower panels show representative immunofluorescence images for the detection (green fluorescence) of STIM-1 (**E**,**H**), Orai1 (**F**,**I**), and Orai3 (**G**,**J**) in cryosections of coronary arteries from young (3 M) (**E**–**G**) and older rats (20 M) (**H**–**J**). The increased immunodetection of Orai1 and Orai3 was noted in vascular preparations from older rats (arrows). Nuclei are counterstained in blue. Magnifications: ×200. Panels (**K**–**M**) show the quantification of immunofluorescence for STIM-1 (**K**), Orai1 (**L**), and Orai3 (**M**), confirming the upregulation of Orai1 and Orai3 proteins in coronary arteries from older rats. Data are expressed as the mean ± S.E.M. of the percentage of average fluorescence obtained in arteries from 3 M rats. N indicates the number of animals from which the sections were collected for the experiments. ** indicates *p* < 0.01 vs. 3 M according to the Mann–Whitney U test.

STIM-1, Orai1, and Orai3 were detected in homogenates of rat coronary arteries. The relative content of STIM-1 was not significantly modified by aging, while relative amounts of Orai1 and Orai3 channels were significantly increased in the coronary vascular tissues from older animals (Figure 5A–D). Immunofluorescence detection of these proteins confirmed the immunoblot results, showing no apparent aging-related modification of STIM-1 (Figure 5E,H), while higher immunoreactivity was observed in older coronary tissues for the Orai1 (Figure 5F,I) and Orai3 channels (Figure 5G,J). Indeed, the increase in the immunodetection of Orai channels is notable in the smooth muscle layer of coronary arteries from old rats (Figure 5I,J). Furthermore, the quantification of the immunofluorescence assays confirmed the increased expression of Orai1 (Figure 5L) and Orai3 (Figure 5M) channels in coronary sections from older rats, while no significant modification by aging was observed for STIM-1 expression (Figure 5K).

## 3. Discussion

The present results show that aging is related to the upregulation of Orai1 and Orai3 calcium channels in rat coronary arteries, which is associated with hypercontractility to serotonin and endothelin and higher sensitivity to STIM/Orai inhibition; this is present in arteries from older male and female rats. The inhibition of this calcium entry system not only reduces agonist-induced contraction but also improves the impaired endothelial vasodilation of coronary vascular bed in older male rats.

The coronary vascular bed is especially sensitive to the progressive impact of aging on vascular function [7]. The present results confirm previous evidence showing the enhancement of serotonin-induced contractions and the impairment of endothelium-dependent vasodilation in coronary arterial segments from older (20-month-old) rats [7,18]. In addition, we report increased contractions induced by the vasoactive peptide endothelin-1 (ET-1) in coronary vessels from older rats, which is consistent with previous evidence [19]. Interestingly, an elevation of intracellular calcium with aging in rat coronary smooth muscle has been associated with age-related hypercontractility [20]. A contribution of the STIM/Orai calcium entry system to this aging-related hypercontractility is supported by inhibitory effects on this alteration by a known STIM/Orai inhibitor, YM-58483 (also known as BTP-2) [21,22,23]. This compound has been described as an inhibitor of Orai channels without specificity among Orai isoforms [24]. However, some evidence suggests a potential interaction with other calcium channels, such as transient receptor potential channels (TRPC) [25]. To confirm the involvement of Orai channels, we evaluated a more specific inhibitor, Synta66, which is considered a specific inhibitor of Orai1 channels [24,25,26]. In coronary vessels from older rats, treatment with Synta66 at concentrations previously shown to inhibit Orai1 [26] was able to significantly reduce 5-HT- and ET-1-induced contractions. This result further supports the involvement of Orai1 channels in the aging-related hypercontractility of coronary vessels.

Immunofluorescence assays indicate that STIM and Orai proteins are localized in both the endothelium and smooth muscle layers of rat coronary arteries. However, the effects on hypercontractility driven by modulating STIM/Orai are not mediated by endothelial affectation, since the inhibitory effects of YM-58483 are retained after removing the endothelium of the coronary vascular segments.

Increasing extracellular potassium concentrations up to depolarizing levels causes the contraction of vascular smooth muscle. These contractions were not enhanced by aging in coronary arteries and, in contrast to the effect observed with agonist-induced contractions, K^+^-induced responses were completely unaffected by the treatment of coronary vessels with YM-58483. This makes sense, since membrane depolarization leads to the opening of L-type calcium channels, allowing for a high calcium inflow [27], which would be less strongly affected by closing Orai1 channels. In fact, the ability of YM-58483 to cause the relaxation of coronary segments precontracted with 5-HT was almost eliminated when the vessels were precontracted with 50 mM K^+^, with the effect being equally strong in those from young rats as in those from older rats; this finding reinforces the idea of the loss of the functional relevance of Orai-mediated calcium entry when the vascular smooth muscle is depolarized. On the other hand, the greater contribution of the STIM/Orai system to vascular aging is again supported by the higher efficiency of YM-58483 in relaxing 5-HT-contracted coronary arteries.

Although the modulation of contractile properties by STIM/Orai would clearly impact coronary function, the ability of YM-58483 to improve the endothelium-dependent vasodilation of coronary arteries from older rats is highly relevant. The aging-induced impairment of endothelial function has been evidenced in varied vascular beds from humans and rats [5,6,7,8]. Moreover, the defective endothelial vasodilation of rat coronary arteries with aging has previously been reported by us and by others [7,18,28,29]. We show that aging leads to a decline not only in NO-dependent endothelial relaxation but also in hyperpolarization-mediated relaxation (EDH, in the presence of NO synthase and COX inhibitors) in rat coronary arteries. Notably, STIM/Orai inhibition improves endothelial relaxation in older coronary vessels under control conditions but also under conditions that only allow for EDH-induced relaxation. However, the lack of an improving effect by YM-58483 on the defective NO-mediated relaxations of older coronary arteries does not necessarily mean that STIM/Orai inhibition is not able to influence NO-mediated endothelial relaxation, since the isolation of the NO component of relaxation involves the depolarization of coronary arteries with K^+^, which, as mentioned above, may prevent Orai-mediated effects. Nevertheless, YM-58483 was able to significantly enhance the relaxation of older coronary arteries when exposed to the small-conductance (SK_Ca_) and intermediate-conductance calcium-activated potassium (IK_Ca_) channels, NS309 [30]. This potentiating effect on NS309-induced relaxations was detected only in older coronary arteries and not in those from young animals, despite the fact that such responses were not impaired by aging. This is relevant to the potential influence of STIM/Orai inhibition on EDH-mediated endothelial relaxation, since endothelial SK_Ca_ and IK_Ca_ channels are thought to be fundamental players in EDH [31,32]. The inhibition of Orai channels could reduce K_Ca_ activation but, in non-excitable cells such as endothelial cells, K_Ca_ can also activate non-voltage gated calcium channels such as Orai [33]. In this sense, the analysis of NS309-induced relaxations in older coronary arteries suggests that Orai inhibition results in the facilitation rather than in the inhibition of responses to IK_Ca_ and SK_Ca_ stimulation, which would be consistent with enhancing the EDH-mediated relaxation of these vessels. This could be relevant in view of evidence showing the upregulated activity of K_Ca_ to be a means of preserving coronary function in animal models of obesity [34,35].

The beneficial effects of STIM/Orai inhibition on coronary endothelial vasodilation are in agreement with the reported negative correlation between the expression of STIM-1 and Orai1 in human mesenteric microvessels and the vasodilatory capacity of these arteries [17]. The improvement of endothelial relaxation in older coronary arteries by STIM/Orai inhibition is not merely due to reduction in precontractile tone, since there were no significant differences in the precontraction level after treatment with YM-58483 at the concentration used in these experiments (10 µM). However, the present results do not allow us to identify the target tissue of STIM/Orai inhibition for enhancing endothelial relaxation in coronary arteries from older rats. Although it is clear that STIM/Orai inhibition in vascular smooth muscle is responsible for reducing aging-related hypercontractility, as follows from the experiments involving endothelium-denuded coronary segments, we cannot neglect the possibility that the Orai inhibitor acts directly on endothelial cells to improve endothelium-dependent relaxations. The lack of an effect of YM-58483 on SNP-induced endothelium-independent relaxations in coronary arteries from old rats might point to an endothelial effect but also could indicate a lack of influence on NO-mediated responses in smooth muscle. Nevertheless, the specific endothelial deletion of STIM-1 in young mice resulted in defective endothelial relaxation [36], suggesting that STIM/Orai inhibition could exert opposing effects when affecting endothelial calcium homeostasis. Further research is required to elucidate the different pathways leading to endothelial vasodilation that are susceptible to the influence of the STIM/Orai system. However, it is clear that the improvement in coronary function in aging could have clinical relevance. CVDs are predominantly considered aging-related diseases because most (>80%) CVD-attributable deaths occur in older individuals [1]. In this sense, coronary dysfunction has been proposed as a therapeutic target in heart failure [37,38]. Specifically, coronary vascular dysfunction is a hallmark of heart failure with a preserved ejection fraction [9]. Thus, strategies focused on improving coronary function in older people could represent valuable therapeutic approaches. In this sense, it is noteworthy that the Orai blockade also reduces agonist-induced contraction in coronary arteries from older female rats, indicating that the mechanism is also relevant for females. This is important since sex differences exist with respect to cardiovascular aging and disease [39]. In fact, most patients with heart failure with a preserved ejection fraction are postmenopausal women [40] and coronary microvascular dysfunction is prevalent in this population [41].

The effects of inhibitors of the STIM/Orai system on coronary function in aging are consistent with the detected upregulation of Orai channels in coronary vascular tissues from older rats. The higher content of both Orai1 and Orai3 channel proteins found in homogenates from older coronary arteries concurs with the immunofluorescent images obtained in arterial segments, showing the intense detection of Orai1 and Orai3 channels in smooth muscle layers of coronary sections from older animals. The altered expression of STIM/Orai proteins with aging has previously been observed in rat and human vascular tissues, but the specific pattern of STIM/Orai upregulation was different depending on the vascular territory assessed. Orai3 channels were upregulated with aging in rat and human corpus cavernosum [16], while STIM-1 and Orai1 were increased in older human mesenteric microvessels and only Orai1 in human aorta [17]. This points to the specific roles of the STIM/Orai system in different vascular beds. Effective inhibition of contractile responses by the specific Orai1 blocker Synta66 would indicate the relevance of Orai1 upregulation in altered coronary function in older rats. However, the lack of specific blockers of Orai3 channels precludes us from understanding the functional role of aging-related Orai3 upregulation. However, it has been proposed that Orai3 could integrate with or otherwise influence STIM-1 and Orai1 activity [42].

The lack of concomitant measurements of intracellular calcium concentrations and functional determinations in coronary arteries may be considered a limitation of this study. Another limitation is that not all experiments were repeated in female animals. Despite the number of questions that are unaddressed in this regard (hormonal regulation, etc.), the present demonstration of the relevance of the STIM/Orai system in coronary vascular aging in female animals may represent a starting point for future research.

## 4. Materials and Methods

### 4.1. Experimental Animals

Young (3-month-old) and older (20-month-old) male Sprague Dawley rats were obtained from the Animal Facilities of the Hospital Universitario Ramón y Cajal and the Hospital Universitario de Getafe. Seven 20-month-old female rats were also used for specific experiments. Animals were maintained in 12 h light/dark cycles with free access to food and water until the experimental procedures were carried out. The rats were bred and let to age in the facilities until reaching the experimental ages. The animal studies were performed in accordance with the Declaration of Helsinki and with the Guide for the Care and Use of Laboratory Animals, as adopted and promulgated by National Institutes of Health, following European regulations; they were approved by the Ethics Committees for Animal Experimentation of the Hospital Universitario de Getafe and the Hospital Universitario Ramón y Cajal (PROEX 005/15; PROEX 215.5/22).

At 3 (*n* = 38 males) and 20 months of age (*n* = 57 males and *n* = 7 females), the rats were weighed and anesthetized with diazepam (5 mg/kg) and ketamine (90 mg/kg). Under deep anesthesia, the hearts were removed, causing the humane death of the animals. Experiments with rats from different ages were intercalated to avoid possible sequence-dependent bias.

### 4.2. Functional Evaluation of Coronary Arteries

The hearts were placed in a Petri dish with Krebs–Henseleit solution (KHS) at 4 °C. The composition of the KHS was (in mM): NaCl 119, KCl 4.6, CaCl_2_ 2.5, MgCl_2_ 1.2, NaHCO_3_ 24.9, glucose 11, KH_2_PO_4_ 1.2, and EDTA 0.027. Left and right coronary arteries were immediately isolated from the excised hearts and carefully cleaned from the surrounding cardiac tissue. Segments of coronary arteries (~1.8 mm long) were set in wire myographs for the recording of the isometric tension, as previously described [7,18]. The vessels were allowed to equilibrate for 30 min in KHS at 37 °C, continuously bubbled with a 95% O_2_/5% CO_2_ mixture to maintain a pH of 7.4. The passive tension and internal circumference of the vascular segments were determined when relaxed in situ under a transmural pressure of 100 mmHg (L_100_). The arteries were then set to an internal circumference equivalent to 90% of L_100_, at which the force development is close to maximal. To assess vessel viability, preparations were then exposed to 125 mM K^+^ (KKHS, equimolar substitution of NaCl for KCl in KHS) and the contractile response was measured. Segments that did not generate a tension above 1 mN were discarded. After a stabilization period, the contraction responses were evaluated by cumulatively adding serotonin (5-HT; 1 nM to 100 µM), endothelin-1 (ET-1, 0.1 to 300 nM) or KCl (10 to 120 mM) to non-pre-contracted coronary artery segments. For the relaxation experiments, vascular segments were contracted with 1–10 µM 5-HT or 50 mM KCl (80% of KKHS-induced contraction, approximately) and relaxation responses were evaluated by cumulative additions of YM-58483 (0.1–30 µM), acetylcholine (ACh, 1 nM to 10 µM), sodium nitroprusside (SNP, 1 nM to 10 µM), or NS309 (0.1–30 µM) to the chambers. In some experiments, the 5-HT-precontracted coronary artery segments were previously incubated with the nitric oxide (NO) synthase (NOS) inhibitor, N^G^-nitro-L-arginine methyl ester (L-NAME; 100 µM), and the cyclooxygenase inhibitor, indomethacin (INDO, 10 µM), while the KCl-contracted arteries were treated with INDO (10 µM). The STIM/Orai inhibitor YM-58483 (20 µM) and a specific blocker of Orai1 channels, Synta66 (10 µM), or the vehicle, were added 30 min before the concentration-response curves started. There were no differences in vascular responses between the right and left coronary arteries (Supplementary Materials Figure S2B).

In specific vascular segments, the endothelium was removed by repeatedly passing a human hair through the lumen of the mounted arterial segments. Then, the viability of the arterial preparation was assessed by exposure to 125 mM K^+^ while the success of the endothelium removal was confirmed by the lack of vasodilation to 10 μM ACh.

### 4.3. Western Blot Assays

Rat coronary arteries were cleaned from the surrounding cardiac tissue and immediately frozen by immersion in liquid nitrogen and stored at −80 °C until the extraction of the proteins. Total protein extracts were obtained by the homogenization of the vascular tissue using a T-PER extraction reagent (Pierce Biotechnology, Inc., Rockford, IL, USA) according to the manufacturer’s recommendations, with the addition of 1x Protease Inhibitor Cocktail (Roche Diagnostics, Indianapolis, IN, USA). Western blot assays were performed as previously described [15,17]. Equal amounts of protein extracts (10 µg) were loaded into a 10% SDS-polyacrylamide gel and resolved via standard SDS-PAGE. Proteins were electrophoretically transferred onto PVDF membranes. The membranes were blocked with 5% skimmed milk in phosphate-buffered saline containing 0.1% Tween 20 for 60 min and tested overnight with specific rabbit or mouse antibodies against STIM-1 (Novus, Littleton, CO, USA, cat.# NBP1-52849, dilution 1:1000), Orai1 (ThermoFisher Scientific, Waltham, MA, USA, cat.# MA5-15776, dilution 1:500), and Orai3 (ThermoFisher, cat.# PA5-20370, dilution 1:500), or a mouse antibody against α-actin (Sigma-Aldrich, Saint Louis, MO, USA, cat.# A2547, dilution 1:2500), which was used as the loading control. The expression of α-actin was not significantly influenced by aging (Supplementary Materials Figure S3). Subsequently, the membranes were incubated with goat anti-mouse (1:5000 dilution; Novus, cat.# NBP2-30347H) or goat anti-rabbit (1:10,000 dilution; Novus, cat.# NB7160) horseradish peroxidase-conjugated secondary antibodies for 1 h at room temperature. Blots were visualized via the ECL detection system (ThermoFisher Scientific). The results were quantified by densitometry using Quantity One/Chemi-Doc ImageLab 6.0 Software (Bio-Rad, Barcelona, Spain). Development without primary antibodies yielded no chemiluminescence signal.

### 4.4. Immunofluorescence Assays

Rat coronary vascular segments were immersed in saccharose (30% *w*/*v*), embedded in an optimal cutting temperature solution (OCT), and stored at −80 °C until the immunofluorescence assays were performed, as previously described [17,18]. Coronary arteries included in the OCT blocks were transversally cut in a cryostat and mounted on FLEX IHC Microscope slides (Dako, Glostrup, Denmark). The 6 µm thick sections were blocked for 30 min with PBS containing 2% BSA and then incubated overnight with rabbit antibodies against STIM-1 (Novus, cat.# NB110-60547, 1:200 dilution), Orai1 (Novus, cat.# NBP1-77289, 1:50 dilution), or Orai3 (Novus, cat.# NBP1-93523, 1:100 dilution) at 4 °C. After washing out in PBS plus 0.05% Triton X-100, the sections were incubated with a secondary Alexa Fluor 488-conjugated goat anti-rabbit antibody (Thermofisher Scientific, cat.# A11008, dilution 1:250) for 45 min at room temperature. Nuclei were counterstained with 300 nM diamidino-2-phenylindole (DAPI, Life Technologies, Alcobendas, Spain) for 5 min at room temperature. Sections were mounted and viewed by fluorescence microscopy (Olympus BX51, Tokyo, Japan). Three to five random images from each specimen were captured, and the fluorescence intensity was quantified and normalized with the DAPI intensity using Image J 1.48i software (McBiophotonics Image J, NIH, Bethesda, MD, USA) after subtracting the background obtained in the negative controls without primary antibodies. An average value for each specimen was obtained. The group corresponding to each specimen was blinded for the investigator capturing and quantifying the immuno-fluorescence images.

### 4.5. Statistical Analysis

Data are expressed as the mean ± standard error. Complete concentration–response curves were compared via a two-factor analysis of variance (ANOVA) test by means of GraphPad Prism 6.0 software (San Diego, CA, USA). This statistical test compares concentration–response curves in their entirety, including all concentrations in the analysis. When multiple curves were compared, Bonferroni’s correction was applied. Other data were compared via Mann–Whitney U tests or Kruskal–Wallis followed by Dunn’s test for multiple comparisons using GraphPad Prism software. pEC_50_ is defined as the –log M of the concentration required to obtain 50% of the maximal relaxation.

## 5. Conclusions

In conclusion, the aging-related upregulation of Orai1 and Orai3 channels in rat coronary arteries may compromise coronary function, which is improved by STIM/Orai inhibition. The recovery of coronary function with STIM-1/Orai inhibition could be a potential therapeutic strategy for treating/preventing age-related CVD.

## Data Availability

All data are available upon request to corresponding author.

## Supplementary Materials

The following supporting information can be downloaded at: https://www.mdpi.com/article/10.3390/ijms241713402/s1.
