# Supplementary material for: Upregulation of Orai Channels Contributes to Aging-Related Vascular Alterations in Rat Coronary Arteries
Source: Int J Mol Sci. 2023 Aug 29;24(17):13402. doi: 10.3390/ijms241713402 (PMC10487684; doi:10.3390/ijms241713402)
Supplement: Supplementary file 1 [file ijms-24-13402-s001.zip › ijms-2530781-supplementary.pdf]

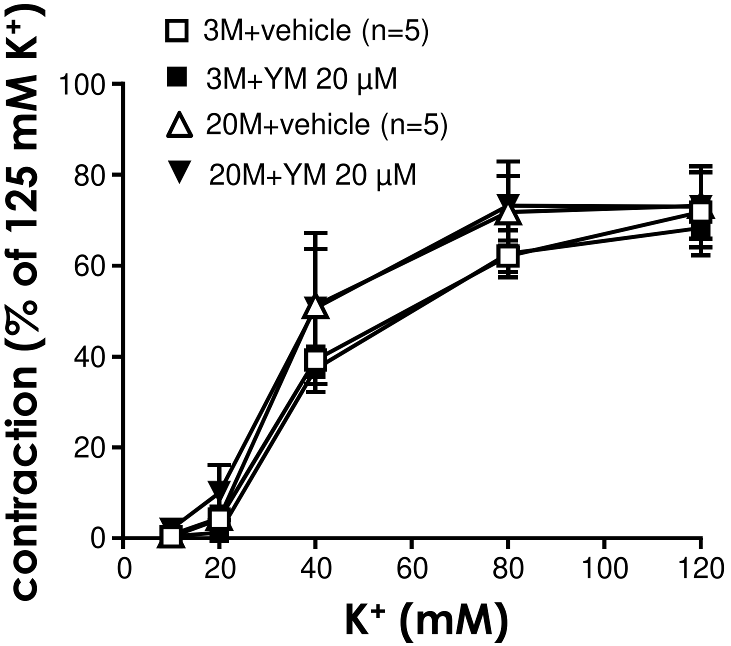

Supplementary Figure S1. STIM/Orai inhibition did not modify K^+-induced contractions in rat coronary arteries. Effects of the treatment with vehicle (0.2% DMSO) or the STIM/Orai inhibitor, YM-58483 (YM, 20 µM) on KCl (10 to 120 mM)-induced contractions in coronary arteries from young (3-months old, 3M) and aged (20-months old, 20M) rats. Data are expressed as mean±S.E.M. of the percentage of 125 mM K^+-induced contraction. n indicates the number of animals used for experiments. No significant differences were obtained by two-factors ANOVA.

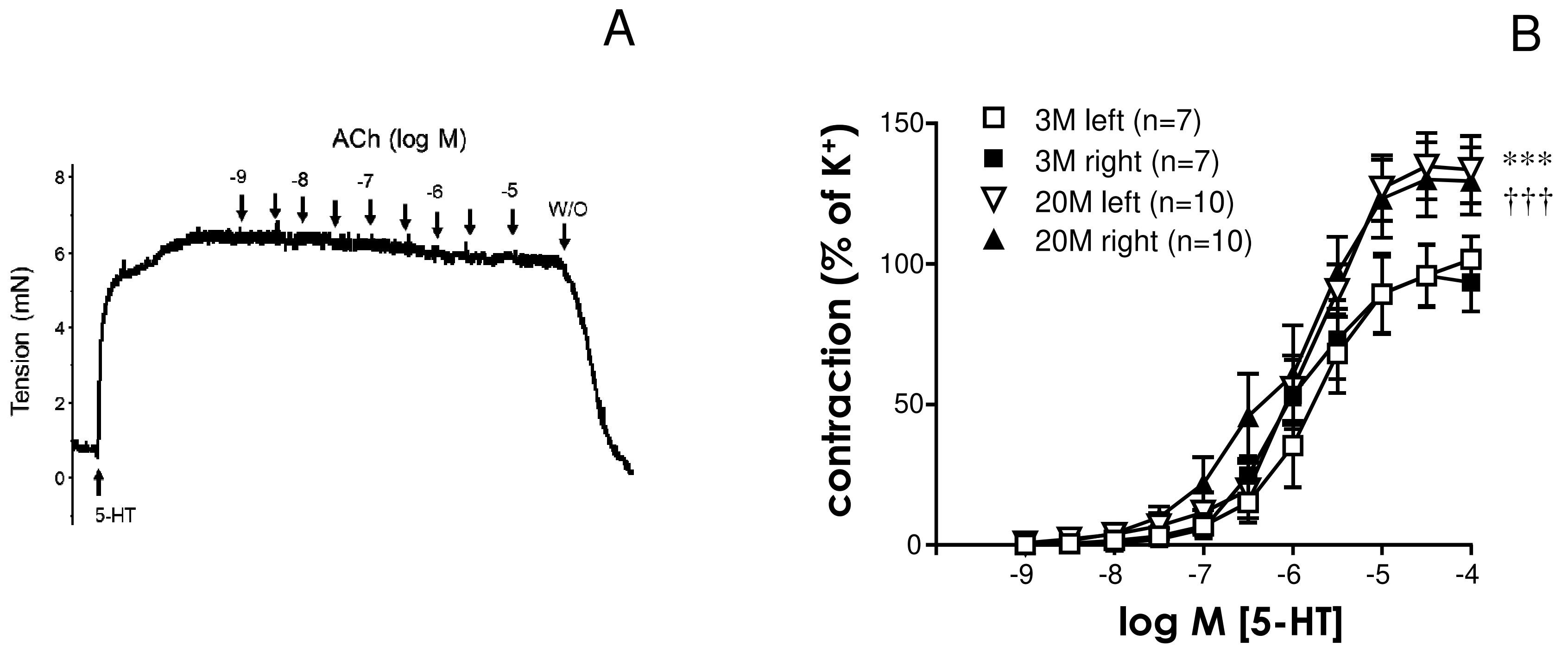

Supplementary Figure S2. Endothelium is required for acetylcholine (ACh)-induced relaxation of rat coronary artery (CA) while left and right coronary arteries display similar contractility in young and aged rats. Panel A shows a representative tracing demonstrating the lack of relaxation to ACh (1 nM to 10 μM) in a CA segment from a young (3-months old, 3M) rat contracted with serotonin (5-HT) and previously denuded of endothelium by repeatedly passing a human hair through the lumen of the arterial segment. W/O indicates washout. Panel B shows contractile responses induced by 5-HT in left and right CA from young (3M) and aged (20-months old, 20M) rats. Data are expressed as mean±S.E.M. of the percentage of 125 mM K_+-induced contraction. n indicates the number of animals used for experiments. No significant differences were obtained by two-factors ANOVA between left and right rat CA while aging-related hypercontractility were maintained when comparing arteries from the same side of the heart. *** $p < 0.001$ vs. respective responses in left CA from 3M rats, ††† $p < 0.001$ vs. respective responses in right CA from 3M rats.

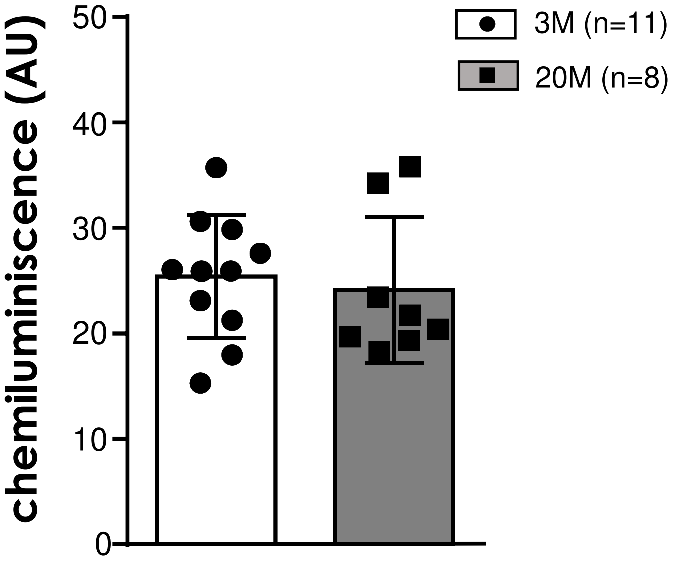

Supplementary Figure S3. Aging did not significantly modify α-actin expression in rat coronary arteries. Cuantification of band densities obtained in immunoblot assays in rat coronary artery homogenates from young (3-months old, 3M) and aged (20-months old, 20M) rats. Data are expressed as mean±SD as well as individual data of band intensity in arbitrary units (AU) of chemiluminiscence. n indicates the number of animals used for experiments. No significant differences were obtained by Mann-Whitney test.
